# Correction to: Abaloparatide effect on forearm bone mineral density and wrist fracture risk in postmenopausal women with osteoporosis

**DOI:** 10.1007/s00198-020-05469-y

**Published:** 2020-06-12

**Authors:** N. B. Watts, G. Hattersley, L. A. Fitzpatrick, Y. Wang, G. C. Williams, P. D. Miller, F. Cosman

**Affiliations:** 1 Mercy Health Osteoporosis and Bone Health Services, Cincinnati, OH USA; 2 Radius Health, Inc., Waltham, MA USA; 3 Colorado Center for Bone Research, Lakewood, CO USA; 4 Columbia University College of Physicians and Surgeons, New York, NY USA

**Correction to: Osteoporosis International (2019) 30:1187–1194**

10.1007/s00198-019-04890-2

The original version of this article, published on 21 March 2019, unfortunately contains some typos in Figs. 2, 3, 4, and Supplemental Fig. 1. The corrected figures are given below.

Fig. 2
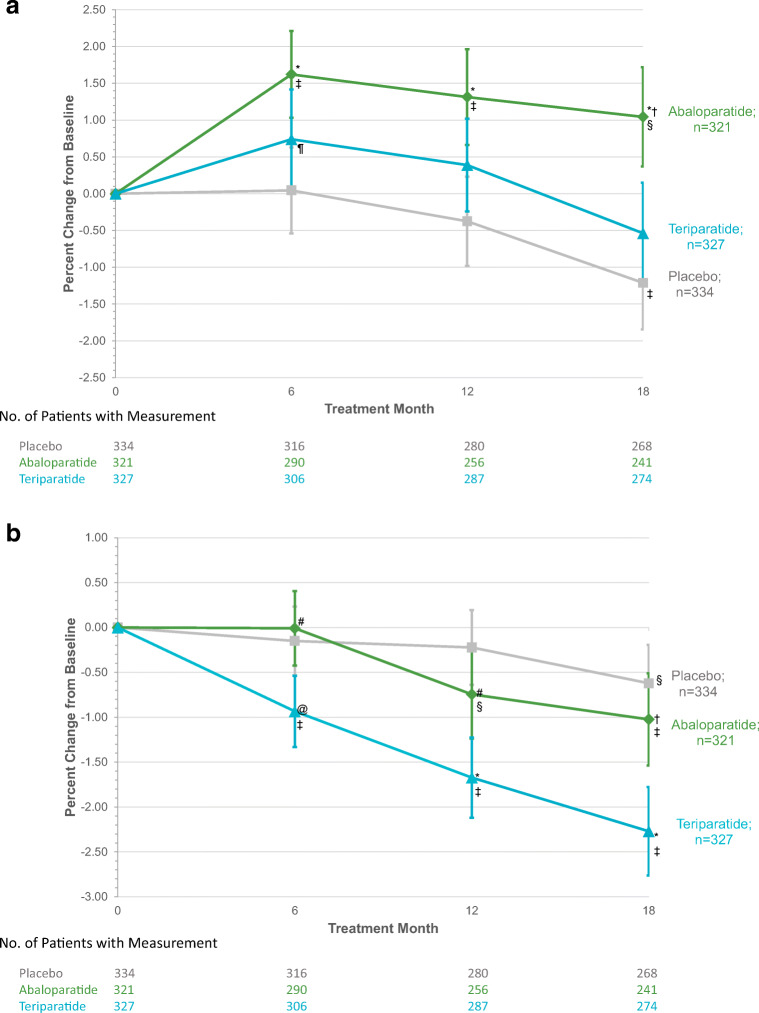


Fig. 3
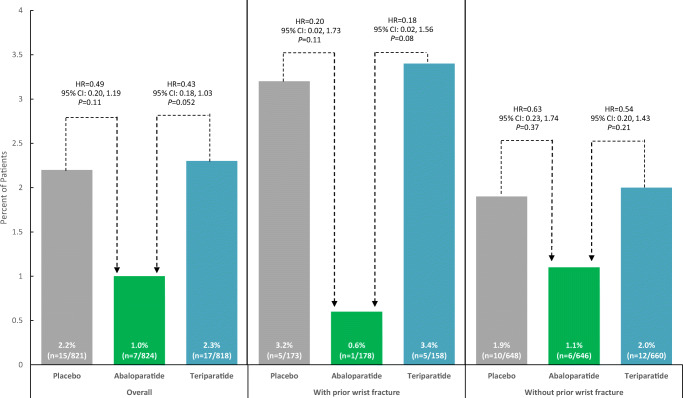


Fig. 4
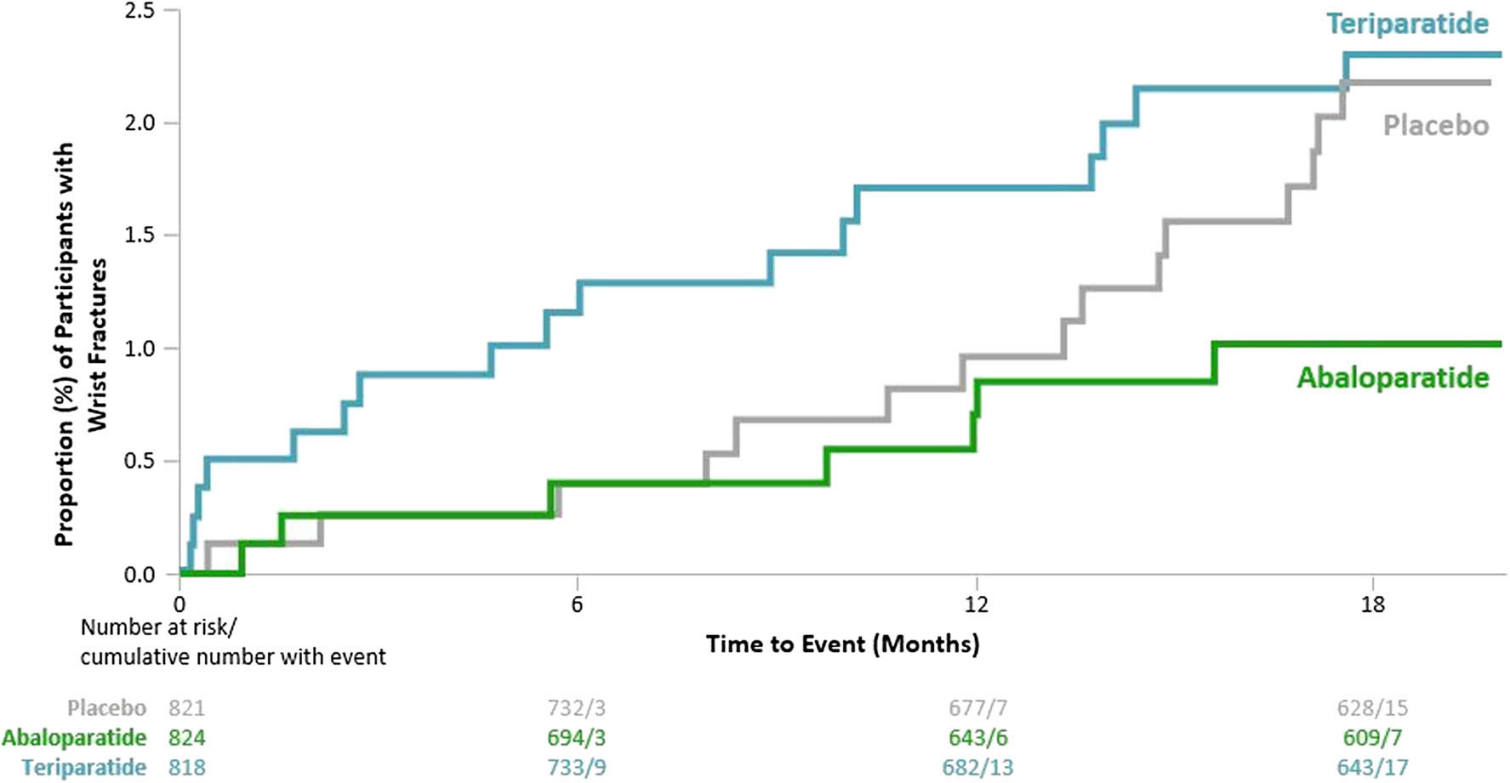


## Electronic supplementary material

ESM 1 (PDF 80.5 kb)

