# Supplementary figures and images for: Correction to: Abaloparatide effect on forearm bone mineral density and wrist fracture risk in postmenopausal women with osteoporosis
Source: Osteoporos Int. 2020 Jun 12;31(8):1603–5. doi: 10.1007/s00198-020-05469-y (PMC7360655; doi:10.1007/s00198-020-05469-y)

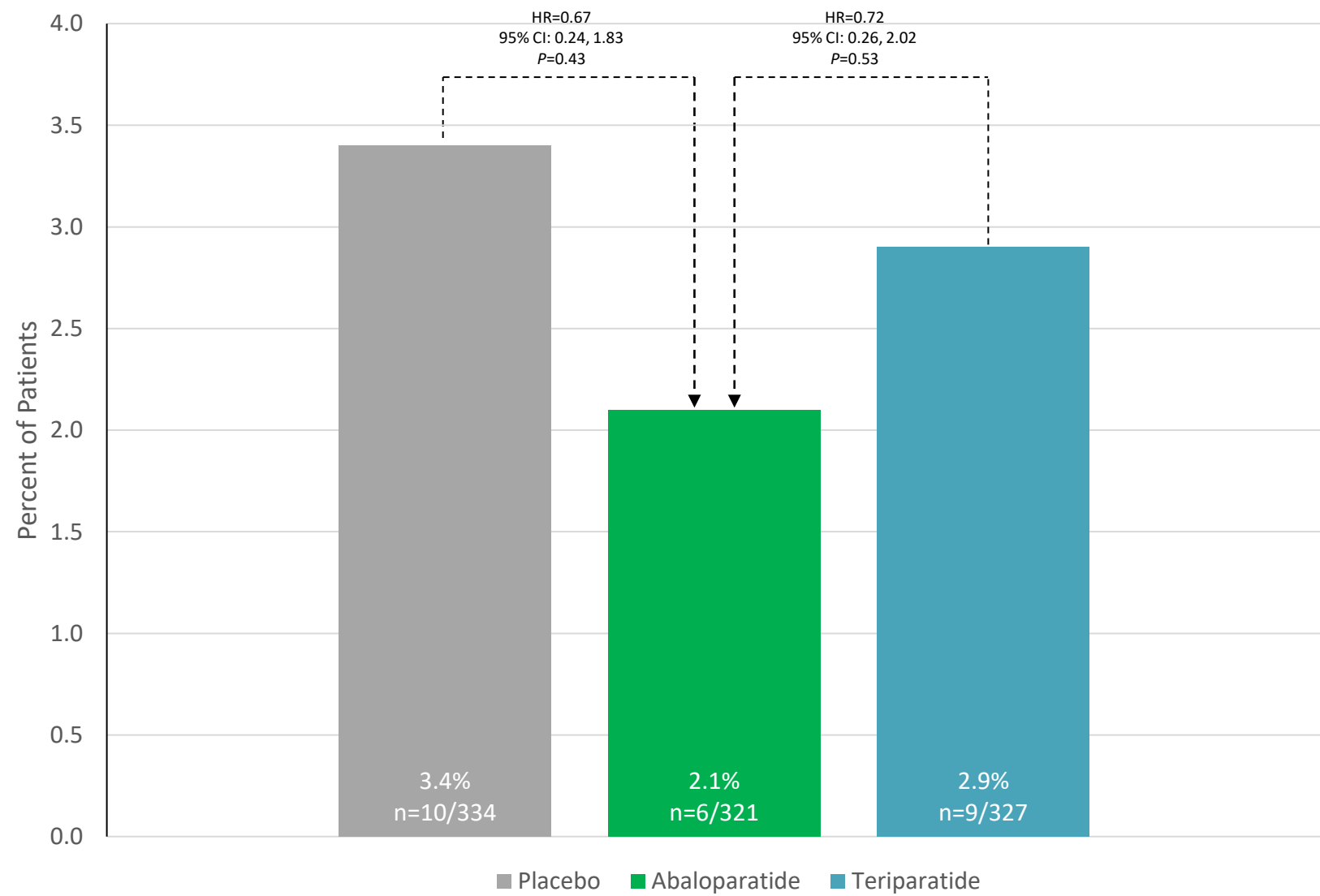

Supplement: Supplementary file 1 — (PDF 80.5 kb) [file 198_2020_5469_MOESM1_ESM.pdf]
